# Supplementary material for: Photon momentum transfer and partitioning: from one to many
Source: Nat Commun. 2025 Jul 1;16:5977. doi: 10.1038/s41467-025-60983-z (PMC12218933; doi:10.1038/s41467-025-60983-z)
Supplement: Supplementary file 1 — Supplementary Information [file 41467_2025_60983_MOESM1_ESM.pdf]

Supplementary Materials for

Photon Momentum Transfer and Partitioning: From One to Many

Xiaodan Mao[1], Hongcheng Ni *[1,2], Kang Lin †[3,4], Pei-Lun He[5,6,7], Hao Liang[8], Sebastian Eckart[9], Feng He[6,7], Kiyoshi Ueda[1,10], Reinhard Dörner[9], and Jian Wu ‡[1,2,11]

[1]State Key Laboratory of Precision Spectroscopy, East China Normal University, Shanghai 200241, China
[2]Collaborative Innovation Center of Extreme Optics, Shanxi University, Taiyuan, Shanxi 030006, China
[3]School of Physics, Zhejiang University, Hangzhou 310058, China
[4]Zhejiang Key Laboratory of Micro-nano Quantum Chips and Quantum Control, Zhejiang University, Hangzhou 310058, China
[5]Max-Planck-Institut für Kernphysik, 69117 Heidelberg, Germany
[6]Key Laboratory for Laser Plasmas (Ministry of Education), School of Physics and Astronomy, Shanghai Jiao Tong University, Shanghai 200240, China
[7]Collaborative Innovation Center for IFSA (CICIFSA), Shanghai Jiao Tong University, Shanghai 200240, China
[8]Max-Planck-Institut für Physik komplexer Systeme, 01187 Dresden, Germany
[9]Institut für Kernphysik, Goethe-Universität Frankfurt, 60438 Frankfurt am Main, Germany
[10]Department of Chemistry, Tohoku University, Sendai 980-8578, Japan
[11]Chongqing Key Laboratory of Precision Optics, Chongqing Institute of East China Normal University, Chongqing 401121, China

S1 Different theoretical approaches and data analysis

Four theoretical approaches have been used to extract the photoelectron momentum distribution (PMD), including time-dependent Schrödinger equation (TDSE), nondipole strong-field approximation (ndSFA), nondipole saddle-point approximation (ndSPA), and nondipole Keldysh–Faisal–Reiss theory (ndKFR).

Shown as in Suppl. Fig. S1 is the comparison of the results of different theoretical methods, where

*hcni@lps.ecnu.edu.cn
†klin@zju.edu.cn
‡jwu@phy.ecnu.edu.cn

row (a), (b), (c) and (d) correspond to TDSE, ndSFA, ndSPA and ndKFR, respectively. Clearly, all four methods give largely identical results, demonstrating the reliability of these theoretical approaches.

Shown in column (1) of Suppl. Fig. S1 is the PMD as a function of the transverse momentum in the polarization plane $p_\perp$ and the lateral momentum in the laser propagation direction p_z. Integration of the PMD along the iso-transverse-energy-$E_\perp$ surfaces gives the blue line in column (2) of Suppl. Fig. S1, which qualitatively follows

$$\langle p_z \rangle_\perp = \frac{E_\perp}{c} + \frac{I_p}{3c}, \tag{S1}$$

where the transverse energy

$$E_\perp = \frac{1}{2m_e} p_\perp^2 = \frac{1}{2m_e} \left(p_x^2 + p_y^2\right). \tag{S2}$$

On the other hand, integration of the PMD along the iso-dressed-energy-E_γ surfaces gives the blue line in column (3) of Suppl. Fig. S1, which quantitatively follows

$$\langle p_z \rangle_\gamma = 2\frac{E_\gamma}{c} - \frac{U_p}{c}, \tag{S3}$$

where the dressed energy

$$E_\gamma = \frac{1}{2m_e} \left[p_\perp^2 + \left(p_z + \frac{U_p}{c} \right)^2 \right]. \tag{S4}$$

To obtain an accurate value of the expectation value of p_z, an adequate integration range covering the essential probability distribution is necessary. Theoretically, the expectation value is obtained by integration over a range of $[-1, 1]$. We have checked the convergence on the choice of the integration range that the results are identical to those obtained with an integration range of $[-2, 2]$. However, the experimentally accessible range in p_z is only $[-0.2, 0.2]$, as indicated by the shaded region between the yellow dashed lines in column (1) of Suppl. Fig. S1. If one carry out the integration over this limited range, the resulting expectation value of p_z would deviate from Eqs. (S1) and (S3), as shown by the yellow lines in columns (2) and (3) of Suppl. Fig. S1.

Due to this limitation in the experimentally accessible range in p_z, one should find another way to extract the expectation value of p_z from the experimentally measured PMD. Remarkably, the peak value of p_z is largely identical to the expectation value of p_z. To demonstrate this point, we fit the distribution

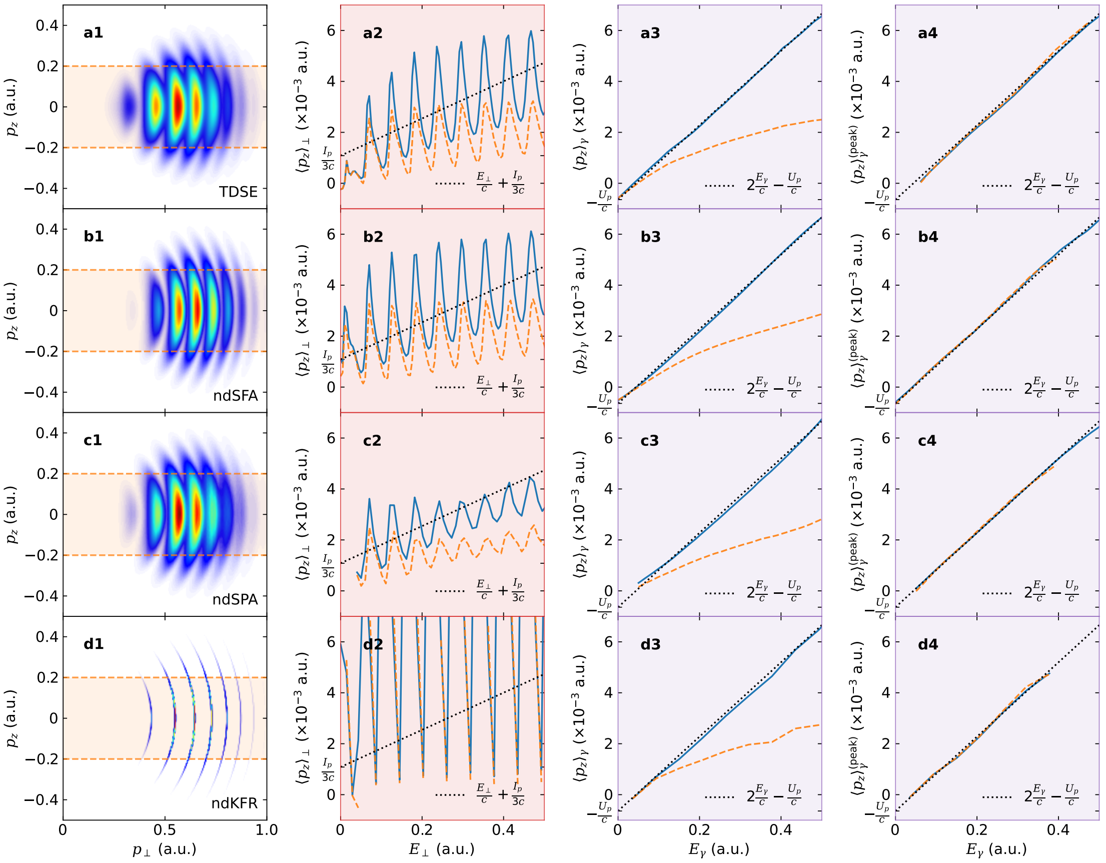

Supplementary Figure S1: **Photoelectron momentum distribution and linear momentum transfer calculated by different theoretical approaches**, including (**a**) TDSE, (**b**) ndSFA, (**c**) ndSPA and (**d**) ndKFR. Column (**1**) is the photoelectron momentum distribution, column (**2**) is the expected linear momentum transfer as a function of the transverse energy $\langle p_z \rangle_\perp$, column (**3**) is the expected linear momentum transfer as a function of the dressed energy $\langle p_z \rangle_\gamma$ and column (**4**) is the peak linear momentum transfer as a function of the dressed energy $\langle p_z \rangle_\gamma^{(\mathrm{peak})}$. Only limited range of p_z is accessible experimentally, as indicated by the orange shaded region between the orange dashed lines in column (**1**). Corresponding linear momentum transfer extracted from this limited range in p_z is shown as orange dashed lines in columns (**2**) to (**4**).

in p_z by a Gaussian function

$$W(p_z) = \beta e^{-\frac{\left(p_z - \langle p_z \rangle^{(\mathrm{peak})}\right)^2}{2\sigma^2}}, \tag{S5}$$

from which the peak value $\langle p_z \rangle^{(\mathrm{peak})}$ can be extracted, as shown as blue lines in column (4) of Suppl. Fig. S1. Most importantly, this peak value stays essentially the same even if one uses limited range in p_z for the fitting. As shown as yellow lines in column (4), $\langle p_z \rangle^{(\mathrm{peak})}$ extracted from the limited fitting range of

$[-0.2, 0.2]$ coincides with that extracted from the full range shown as blue lines. Both follow Eq. (S3) closely.

In summary, all four theoretical approaches give largely identical results in the PMD and the linear momentum transfer. Iso-transverse-energy integration leads to Eq. (S1) while iso-photonic-energy integration results in Eq. (S3). Due to limited detection range in p_z experimentally, one should use the peak value of p_z, which largely coincides with the expectation value of p_z, since the distribution of linear momentum transfer is essentially Gaussian shaped.

S2 Focal volume effect

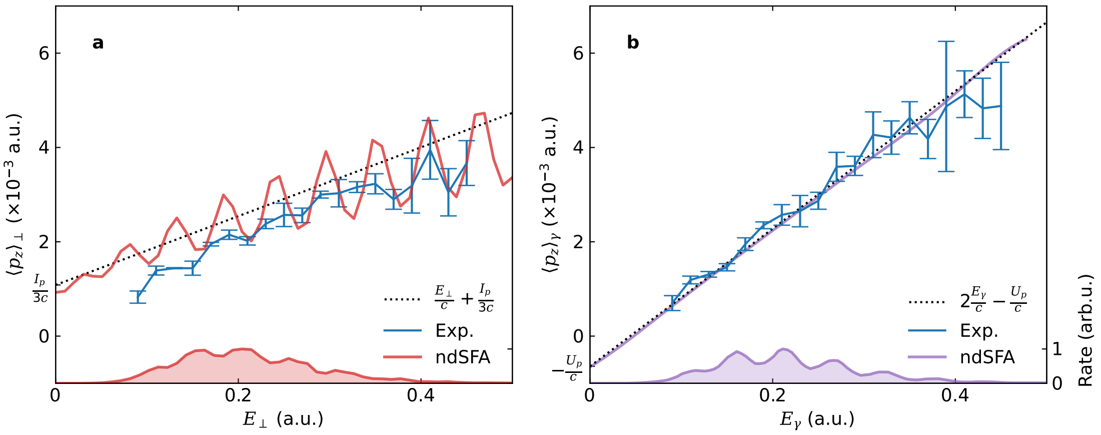

Supplementary Figure S2: Linear momentum transferred and the spectrum of the above-threshold ionization as a function of the **a** transverse energy $E_\perp$ and **b** dressed energy E_γ after considering focal volume averaging.

We further consider the effect of the focal volume averaging on the dependence of $\langle p_z \rangle$. To this end, the final PMD is obtained by a weighted summation over individual PMDs obtained with a number of laser intensities using ndSFA. The relative weight of a specific intensity is given by [1]

$$dV/dI \propto (I_0 + 2I)\sqrt{I_0 - I}/I^{5/2}, \tag{S6}$$

where I_0 is the peak intensity at the laser focus.

After focal volume averaging, the expected linear momentum transfer is shown in Suppl. Fig. S2.

Clearly, the focal volume effect does not alter our conclusions that, in the present framework of photon momentum transfer, for each photon absorbed above the ionization threshold, twice of the photon momentum is transferred to the photoelectron.

S3 Analysis of $\langle p_z \rangle_\perp$

In Suppl. Fig. 2**a** of the main text, the experimental results for $\langle p_z \rangle_\perp$ consistently fall below the theoretical curve $E_\perp/c + I_p/3c$. This discrepancy arises because the theoretical curve is based on the adiabatic approximation and neglects the influence of the prefactor in the ionization rate derived from ndSPA. To address this, we have derived the average linear momentum transfer in the full setting, as previously reported in Ref. [2]:

$$\begin{aligned}
\langle p_z \rangle_\perp^{(\mathrm{NA},\, \alpha_Z)} &= \frac{\langle p_\perp^2 \rangle}{2m_e c} - \frac{\langle v_\perp^2 \rangle}{2m_e c} + \left[1 - \hbar\sqrt{\frac{e^2}{m_e}\frac{2\alpha_Z F_0}{(2I_p)^{3/2}}}\right]\frac{2m_e I_p + \langle v_\perp^2 \rangle}{6m_e c} \\
&= \frac{E_\perp}{c} - \frac{\langle v_\perp^2 \rangle}{2m_e c} + \left[1 - \hbar\sqrt{\frac{e^2}{m_e}\frac{2\alpha_Z F_0}{(2I_p)^{3/2}}}\right]\frac{2m_e I_p + \langle v_\perp^2 \rangle}{6m_e c} \\
&= \frac{\Delta E_\perp}{c} + \delta\frac{\tilde{I}_p}{3c},
\end{aligned} \tag{S7}$$

where

$$\Delta E_\perp = E_\perp - E_{\perp 0} = E_\perp - \frac{1}{2m_e}\langle v_\perp^2 \rangle \tag{S8}$$

denotes the energy absorption during the continuum motion after tunneling,

$$\tilde{I}_p = I_p + E_{\perp 0} = I_p + \frac{1}{2m_e}\langle v_\perp^2 \rangle \tag{S9}$$

is the effective ionization potential accounting for the initial kinetic energy at the tunnel exit, and

$$\delta = 1 - \hbar\sqrt{\frac{e^2}{m_e}\frac{2\alpha_Z F_0}{(2I_p)^{3/2}}} = 1 - \hbar\sqrt{\frac{e^2}{m_e}\frac{2\left(1 + Z/\sqrt{(2I_p)/\left[2I_p^{(\mathrm{H})}\right]}\right)F_0}{(2I_p)^{3/2}}} \tag{S10}$$

roots in the prefactor in the ndSPA ionization rate. For nonadiabatic tunneling, the influence of the initial kinetic energy $E_{\perp 0}$ at the tunnel exit cannot be neglected [2]:

$$E_{\perp 0} = \frac{1}{2m_e}\langle v_\perp^2 \rangle = \frac{1}{2m_e}\langle v_\perp \rangle^2 + \hbar\sqrt{\frac{e^2}{m_e}\frac{F_0}{4\sqrt{2I_p}}} = \frac{1}{2m_e}\left(\frac{m_e}{e}\frac{I_p}{3A_0}\right)^2 + \hbar\sqrt{\frac{e^2}{m_e}\frac{F_0}{4\sqrt{2I_p}}}. \tag{S11}$$

If we assume adiabatic tunneling with $E_{\perp 0} \to 0$ and neglect the influence of the prefactor by setting $\alpha_Z \to 0$ (or $\delta \to 1$), we obtain the commonly used simplified formula:

$$\langle p_z \rangle_\perp^{(\mathrm{A},\, \alpha_Z=0)} = \frac{E_\perp}{c} + \frac{I_p}{3c}. \tag{S12}$$

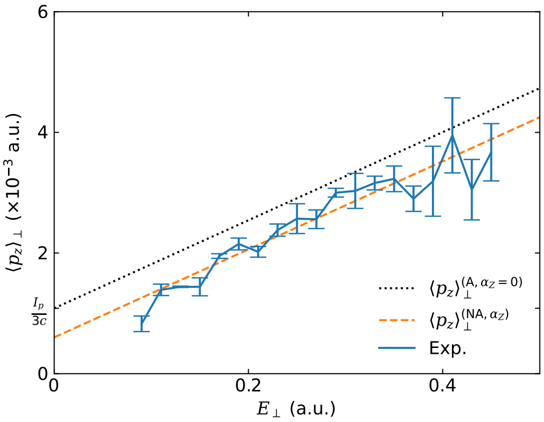

Supplementary Figure S3: Linear momentum transfer $\langle p_z \rangle_\perp$ as a function of the transverse energy $E_\perp$. The black dotted line represents Eq. (S12), the orange dashed line represents Eq. (S7), and the blue line represents the experimental results.

Suppl. Fig. S3 illustrates the linear momentum transfer $\langle p_z \rangle_\perp$ as a function of the transverse energy $E_\perp$. The black dashed line represents the simplified formula [Eq. (S12)], while the orange dashed line represents the full expression [Eq. (S7)]. The blue line corresponds to the experimental results. As evident in Suppl. Fig. S3, the orange dashed curve, which accounts for nonadiabatic effects and the ndSPA prefactor, lies consistently lower than the black dashed curve. This downward shift better aligns with the experimental results, highlighting the importance of considering nonadiabatic effects and the prefactor in the theoretical model.

S4 Experimental data for different laser propagation directions

To mitigate systematic errors, we employed two counter-propagating laser pulses to create standing waves (SW). In this configuration, the nondipole effect is expected to vanish, allowing for precise calibration of the momentum zero in the laser propagation direction. During the measurement of linear momentum transfer, one of the two counter-propagating lasers is turned off.

Suppl. Fig. S4 displays the experimental measurements for laser propagation along the $+\hat{\mathbf{e}}_z$ and $-\hat{\mathbf{e}}_z$ directions, along with the corresponding SW data. The data are divided into two subsets based on the electron momentum along the polarization direction ($\hat{\mathbf{e}}_x$): one subset with positive p_x and the other with negative p_x, corresponding to positive and negative energies, respectively.

The results demonstrate that the SW curve is positioned between the "forward" and "backward" curves, although it does not align perfectly with zero due to the inhomogeneity of the detector. Additionally, the positive and negative energy curves exhibit similar trends, which can be attributed to the symmetry of the laser polarization direction.

These observations validate the integrity of our experimental data. To further eliminate systematic errors, we processed the data by subtracting the "backward" curve from the "forward" curve and then dividing the result by two. The final processed results are presented in Fig. 2 of the main text.

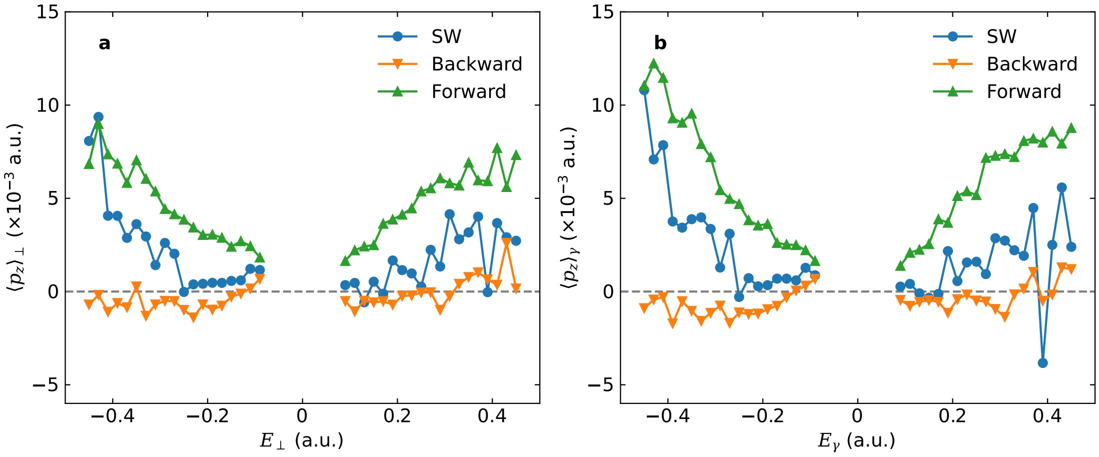

Supplementary Figure S4: Linear momentum transfer as a function of the **a** transverse energy $E_\perp$ and **b** dressed energy E_γ for the forward-propagating (along $+\hat{\mathbf{e}}_z$ direction), backward-propagating (along $-\hat{\mathbf{e}}_z$ direction) and standing wave (SW) cases. The positive and negative energies represent those electrons with positive and negative momentum along the polarization direction ($\hat{\mathbf{e}}_x$).

References

[1] Liu, M.-M. *et al.* Effects of orbital and Coulomb potential in strong-field nonadiabatic tunneling ionization of atoms. *Phys. Rev. A* **96**, 043410 (2017).

[2] Ni, H. *et al.* Theory of subcycle linear momentum transfer in strong-field tunneling ionization. *Phys. Rev. Lett.* **125**, 073202 (2020).
